# What is a Pharmacist: Opinions of Pharmacy Department Academics and Community Pharmacists on Competences Required for Pharmacy Practice

**DOI:** 10.3390/pharmacy4010012

**Published:** 2016-02-01

**Authors:** Jeffrey Atkinson, Kristien de Paepe, Antonio Sánchez Pozo, Dimitrios Rekkas, Daisy Volmer, Jouni Hirvonen, Borut Bozic, Agnieska Skowron, Constantin Mircioiu, Annie Marcincal, Andries Koster, Keith Wilson, Chris van Schravendijk, Jamie Wilkinson

**Affiliations:** 1 Pharmacolor Consultants Nancy, 12 rue de Versigny, Villers 54600, France; 2 Pharmacy Faculty, Vrije Universiteit Brussel, Laarbeeklaan 103, Brussels 1090, Belgium; kdepaepe@vub.ac.be; 3 Faculty of Pharmacy, University of Granada (UGR), Campus Universitario de la Cartuja s/n, Granada 18701, Spain; sanchezp@ugr.es; 4 School of Pharmacy, National and Kapodistrian University Athens, Panepistimiou 30, Athens 10679, Greece; rekkas@pharm.uoa.gr; 5 Pharmacy Faculty, University of Tartu, Nooruse 1, Tartu 50411, Estonia; daisy.volmer@ut.ee; 6 Pharmacy Faculty, University of Helsinki, Yliopistonkatu 4, P.O. Box 33-4, Helsinki 00014, Finland; jouni.hirvonen@helsinki.fi; 7 Faculty of Pharmacy, University of Ljubljana, Askerceva cesta 7, Ljubljana 1000, Slovenia; Borut.Bozic@ffa.uni-lj.si; 8 Pharmacy Faculty, Jagiellonian University, UL, Golebia 24, Krakow 31-007, Poland; askowron@cm-uj.krakow.pl; 9 Pharmacy Faculty, University of Medicine and Pharmacy “Carol Davila” Bucharest, Dionisie Lupu 37, Bucharest 020021, Romania; constantin.mircioiu@yahoo.com; 10 European Association of Faculties of Pharmacy, Faculty of Pharmacy, Université de Lille 2, Lille 59000, France; annie.marcincal@pharma.univ-lille2.fr; 11 European Association of Faculties of Pharmacy, Dept. Pharmaceutical Sciences, Utrecht University, PO Box 80082, Utrecht 3508 TB, The Netherlands; A.S.Koster@uu.nl; 12 Applied Health Research Unit, School of Life and Health Sciences, Aston University, Birmingham B4 7ET, UK; k.a.wilson@aston.ac.uk; 13 MEDINE2, Medical Faculty, Vrije Universiteit Brussel, Laarbeeklaan 103, Brussels 1090, Belgium; chrisvs@vub.ac.be; 14 Pharmaceutical Group of the European Union (PGEU), Rue du Luxembourg 19, Brussels 1000, Belgium; j.wilkinson@pgeu.eu

**Keywords:** education, academic, practice

## Abstract

This paper looks at the opinions of 241 European academics (who provide pharmacy education), and of 258 European community pharmacists (who apply it), on competences for pharmacy practice. A proposal for competences was generated by a panel of experts using Delphi methodology. Once finalized, the proposal was then submitted to a large, European-wide community of academics and practicing pharmacists in an additional Delphi round. Academics and community pharmacy practitioners recognized the importance of the notion of patient care competences, underlining the nature of the pharmacist as a specialist of medicines. The survey revealed certain discrepancies. Academics placed substantial emphasis on research, pharmaceutical technology, regulatory aspects of quality, *etc.*, but these were ranked much lower by community pharmacists who concentrated more on patient care competences. In a sub-analysis of the data, we evaluated how perceptions may have changed since the 1980s and the introduction of the notions of competence and pharmaceutical care. This was done by splitting both groups into respondents < 40 and > 40 years old. Results for the subgroups were essentially statistically the same but with some different qualitative tendencies. The results are discussed in the light of the different conceptions of the professional identity of the pharmacist.

## 1. Introduction

There have been a number of changes in pharmacy education over the past 20 years starting firstly with the introduction of the concept of “competence for practice”. In 1974 there were two publications in pharmacy education with the word “competence” in the title, in 2013 there were 66 [1]. Numerous frameworks have been proposed for the development and monitoring of pharmacy practice based such competence frameworks [2,3,4,5]. Several studies have shown that such competence frameworks can be successfully used to improve performance in community pharmacists [6,7]. However, little attention has been paid to the use of, and attitudes to, competence frameworks in pre-graduate, pre-registration university education. Therefore, in order to assess the relevance of pharmacy competencies, this study used the PHAR-QA “Quality Assurance in European Pharmacy Education and Training” [8] project to look at the attitudes of academics and practicing community pharmacists to the competences required for pharmacy practice.

A second change in pharmacy education over the past 20 years concerns the notion of pharmaceutical care. Pharmaceutical care is the responsible provision of drug therapy for the purpose of achieving outcomes that improve a patient’s quality of life. It is englobed in a wider notion of patient care that refers to services rendered by healthcare professionals, and non-professionals under their supervision, for the benefit of the patient.

The number of articles published that have “pharmaceutical care” in the title has risen from one in 1960 to 210 in 2008 (see reference to Web of Science cited previously). This rise is similar to the rise in the interest in the notion of competence described in the previous paragraph. In a *post-hoc* sub-analysis, therefore, we looked at attitudes to competences in age sub-groups (< 40 and > 40 years old). It could be expected that the younger age subgroup will have been much more exposed to the changes outlined above than the older age subgroup.

## 2. Experimental Section

To evaluate academics’ attitudes to competences we asked academics in European pharmacy departments to rank 68 competences for pharmacy practice. Results were compared to those obtained from practicing community pharmacists.

The numbers of respondents are statistically representative of the overall European populations (academics 10,000, community pharmacists 400,000 [9]). Respondents came from 36 different countries and although not representing a homogenous population, it was a representative selection including all significant subgroups (age, profession, region, *etc.*).

The methodology employed has been described in detail elsewhere (PHAR-QA reference cited above). The main steps are shown in Table 1.

In order to check for any possible evolution in attitudes, in a sub-analysis we compared results from 2 different age subgroups: < 40 and > 40 years of age. It is to be expected that the younger age subgroup would have been more exposed to the introduction of the concepts of competence and patient care than the older age subgroup.

GraphPad software was used for statistical analysis (ref).

**Table 1:** The PHAR-QA “Quality Assurance in European Pharmacy Education and Training” study methodology.

Step	Phase
1	A competence framework based on published frameworks for healthcare specialists was produced by 3 rounds of a Delphi process with an expert panel consisting of the authors of this paper, 10/13 of whom practice as pharmacists (in addition to their academic employment).
2	The competences for practice produced after the 3rd Delphi round, were ranked by a large, European-wide population of academics and community pharmacists using the PHAR-QA *surveymonkey* [10] questionnaire. Respondents came from 36/49 countries of the European Higher Education Area [11].
3	The first 6 questions were on the profile of the respondent (age, duration of practice, country of residence, current occupation (academic, community pharmacist)).
4	Questions 7 through 19 asked respondents to rank 68 competences arranged in 13 clusters of (see annex). Questions in clusters 7 (numbering succeeding the 6th question of the responder profile) through 11 were concerned with personal competences, and in clusters 12 through 19 with patient care competences.
5	Respondents were asked to rank the proposals for competences on a 4-point Likert scale:
	1. Not important = Can be ignored;
	2. Quite important = Valuable but not obligatory;
	3. Very important = Obligatory, with exceptions depending upon field of pharmacy practice;
	4. Essential = Obligatory.
	There was also a “cannot rank” possibility as well as the possibility of leaving an answer blank.
6	Ranking scores were calculated as (frequency rank 3 + frequency rank 4) as % of total frequency; this represents the percentage of respondents that considered a given competence as “obligatory”.
	This calculation is based on that used by the MEDINE (*Medical Education in Europe*) consortium that ranked the competences for medical practice [12].
7	Leik ordinal consensus [13] was calculated as an indication of the dispersion of the data. Responses for consensus were arbitrarily classified as: < 0.2 poor, 0.21–0.4 fair, 0.41–0.6 moderate, 0.61–0.8 substantial, > 0.81 good, as in the MEDINE study.
8	The statistical significance of differences amongst groups was estimated from the chi-square test on the ranking frequencies; a significance level of 5% was chosen.
9	Respondents could also comment on their ranking. An attempt was made to analyze comments using the NVivo10 program [14] for the semi-quantitative analysis of unstructured data. In this case, the numbers were too small to draw significant conclusions (results not shown).

## 3. Results and Discussion

The overall rankings by academics and community pharmacists, given in Table 2, were similar.

**Table 2:** Overall distribution (*n* = 68 competences) of rankings by academics and community pharmacists.

Ranks	Academics		Community Pharmacists	
Number of respondents	241		258	
Theoretical number of replies	16,388 (= 241 respondents × 68 competences)		17,544 (= 258 × 68)	
Rank	Number	%	Number	%
4	5821	38.6	6643	37.9
3	6005	39.6	6002	34.2
2	2982	19.7	3076	17.5
1	366	4.6	608	3.5
Cannot rank + blanks	1214	8.0	1215	6.9
Score (%)	= ((5821 + 6005)/15,174) × 100) = 77.9		= [(6643 + 6002)/16,3029] × 100 = 77.4	
Leik ordinal consensus	0.58		0.55	

Seven to eight percent of respondents in both groups were not able to rank all competences. This suggests that the vast majority of respondents considered they had sufficient experience to reply to all the questions asked.

As judged from the Leik ordinal consensus values, dispersion was low. Leik ordinal consensus for rankings of individual competences ranged from 0.51 to 0.68 for academics, and from 0.42 to 0.71 for community pharmacists. This suggests that opinions in both groups were relatively homogeneous, and that subgroups with distributions of responses concerning the 68 competences significantly different from that of the overall group do not exist. Similar values for ordinal consensus were reported by the MEDINE “Medical Education in Europe” consortium. It further suggests that there are no differences between age subgroups.

Scores for individual competences, given in Table 3, differed.

**Table 3:** Ranking of competences (score of validated competences as important (rank 3 or 4), %) by academics and community pharmacists (*n*: sequential numbering).

Cluster	*n*	Competence	Academics	Community Pharmacists
Cluster 7. Personal competences: learning and knowledge.	1	Ability to identify learning needs and to learn independently (including continuous professional development (CPD)).	93.7	89.8
	2	Analysis: ability to apply logic to problem solving, evaluating pros and cons and following up on the solution found.	94.5	91.1
	3	Synthesis: capacity to gather and critically appraise relevant knowledge and to summarize the key points.	92.8	87.9
	**4**	**Capacity to evaluate scientific data in line with current scientific and technological knowledge.**	**87.3**	**75.8**
	5	Ability to interpret preclinical and clinical evidence-based medical science and apply the knowledge to pharmaceutical practice.	81.2	75.9
	**6**	**Ability to design and conduct research using appropriate methodology.**	**65.4**	**40.2**
	7	Ability to maintain current knowledge of relevant legislation and codes of pharmacy practice.	86.3	91.7
Cluster 8. Personal competences: values.	8	Demonstrate a professional approach to tasks and human relations.	91.5	94.5
	9	Demonstrate the ability to maintain confidentiality.	92.3	95.3
	**10**	**Take full personal responsibility for patient care and other aspects of one’s practice.**	**88.3**	**94.8**
	11	Inspire the confidence of others in one's actions and advice.	83.8	88.8
	12	Demonstrate high ethical standards.	95.3	95.2
Cluster 9. Personal competences: communication and organizational skills.	13	Effective communication skills (both orally and written).	93.5	94.8
	14	Effective use of information technology.	83.8	86.1
	15	Ability to work effectively as part of a team.	83.3	89.2
	16	Ability to identify and implement legal and professional requirements relating to employment (e.g., for pharmacy technicians) and to safety in the workplace.	77.9	81.0
	17	Ability to contribute to the learning and training of staff.	79.6	82.5
	**18**	**Ability to design and manage the development processes in the production of medicines.**	**60.0**	**43.2**
	19	Ability to identify and manage risk and quality of service issues.	76.1	79.2
	20	Ability to identify the need for new services.	61.8	64.5
	21	Ability to communicate in English and/or locally relevant languages.	79.6	74.1
	22	Ability to evaluate issues related to quality of service.	71.0	77.9
	**23**	**Ability to negotiate, understand a business environment and develop entrepreneurship.**	**46.4**	**64.1**
Cluster 10. Personal competences: knowledge of different areas of the science of medicines.	24	Plant and animal biology.	31.1	39.3
	25	Physics.	25.6	21.7
	26	General and inorganic chemistry.	45.6	43.9
	**27**	**Organic and medicinal/pharmaceutical chemistry.**	**80.2**	**66.0**
	**28**	**Analytical chemistry.**	**60.0**	**41.9**
	29	General and applied biochemistry (medicinal and clinical).	74.2	68.8
	**30**	**Anatomy and physiology; medical terminology.**	**75.8**	**88.7**
	31	Microbiology.	67.0	72.2
	32	Pharmacology including pharmacokinetics.	95.6	94.7
	33	Pharmacotherapy and pharmaco-epidemiology.	92.5	94.3
	**34**	**Pharmaceutical technology including analyses of medicinal products.**	**89.0**	**62.0**
	35	Toxicology.	84.4	74.0
	36	Pharmacognosy.	52.9	66.5
	37	Legislation and professional ethics.	88.8	89.5
Cluster 11. Personal competences: understanding of industrial pharmacy.	**38**	**Current knowledge of design, synthesis, isolation, characterization and biological evaluation of active substances.**	**57.5**	**41.7**
	**39**	**Current knowledge of good manufacturing practice (GMP) and of good laboratory practice (GLP).**	**75.4**	**59.4**
	**40**	**Current knowledge of European directives on qualified persons (QPs).**	**59.2**	**43.7**
	**41**	**Current knowledge of drug registration, licensing and marketing.**	**72.1**	**55.7**
	42	Current knowledge of good clinical practice (GCP).	68.2	64.5
Cluster 12. Patient care competences: patient consultation and assessment.	43	Ability to perform and interpret medical laboratory tests.	65.3	65.5
	44	Ability to perform appropriate diagnostic or physiological tests to inform clinical decision making e.g., measurement of blood pressure.	64.5	73.6
	45	Ability to recognize when referral to another member of the healthcare team is needed because a potential clinical problem is identified (pharmaceutical, medical, psychological or social).	89.1	91.7
Cluster 13. Patient care competences: need for drug treatment	46	Retrieval and interpretation of relevant information on the patient's clinical background.	79.3	84.0
	47	Retrieval and interpretation of an accurate and comprehensive drug history if and when required.	89.4	91.5
	48	Identification of non-adherence and implementation of appropriate patient intervention.	85.8	86.8
	49	Ability to advise to physicians and—in some cases—prescribe medication.	80.7	87.6
Cluster 14. Patient care competences: drug interactions.	50	Identification, understanding and prioritization of drug–drug interactions at a molecular level (e.g., use of codeine with paracetamol).	91.8	91.6
	51	Identification, understanding, and prioritization of drug–patient interactions, including those that preclude or require the use of a specific drug (e.g., trastuzumab for treatment of breast cancer in women with HER2 overexpression).	87.7	89.7
	52	Identification, understanding, and prioritization of drug–disease interactions (e.g., NSAIDs in heart failure).	94.5	96.6
Cluster 15. Patient care competences: provision of drug product.	**53**	**Familiarity with the bio-pharmaceutical, pharmacodynamic and pharmacokinetic activity of a substance in the body.**	**90.8**	**81.2**
	54	Supply of appropriate medicines taking into account dose, correct formulation, concentration, administration route and timing.	96.3	94.9
	55	Critical evaluation of the prescription to ensure that it is clinically appropriate and legal.	94.1	94.0
	56	Familiarity with the supply chain of medicines and the ability to ensure timely flow of drug products to the patient.	78.6	84.6
	57	Ability to manufacture medicinal products that are not commercially available.	69.0	60.5
Cluster 16. Patient care competences: patient education.	58	Promotion of public health in collaboration with other actors in the healthcare system.	75.1	82.6
	59	Provision of appropriate lifestyle advice on smoking, obesity, *etc.*	71.0	80.9
	60	Provision of appropriate advice on resistance to antibiotics and similar public health issues.	89.4	93.1
Cluster 17. Patient care competences: provision of information and service.	61	Ability to use effective consultations to identify the patient's need for information.	81.1	90.9
	62	Provision of accurate and appropriate information on prescription medicines.	89.3	94.4
	63	Provision of informed support for patients in selection and use of non-prescription medicines for minor ailments (e.g., cough remedies...).	89.4	94.0
Cluster 18. Patient care competences: monitoring of drug therapy.	64	Identification and prioritization of problems in the management of medicines in a timely manner and with sufficient efficacy to ensure patient safety.	87.9	93.0
	65	Ability to monitor and report to all concerned in a timely manner, and in accordance with current regulatory guidelines on Good Pharmacovigilance Practices (GVPs), Adverse Drug Events and Reactions (ADEs and ADRs).	80.9	83.4
	66	Undertaking of a critical evaluation of prescribed medicines to confirm that current clinical guidelines are appropriately applied.	81.6	80.6
Cluster 19. Patient care competences: evaluation of outcomes.	67	Assessment of outcomes on the monitoring of patient care and follow-up interventions.	73.7	79.0
	68	Evaluation of cost effectiveness of treatment.	57.7	61.2

Notes: Competences in bold are those showing a statistically significant difference in distribution of rankings between groups (chi-square, *p* < 0.05).

Both groups scored high for patient care competences (clusters 12–19). For competences linked to drug research, development and production (clusters 7 and 11), results differed.

In cluster 7 (learning and knowledge), ranks were high (> 80% for 6 out of 7 competences). Scores were lower for competence 6 (ability to design and conduct research using appropriate methodology) and fell to 40% for community pharmacists. The latter proved to be more attached to competencies connected with patient care, though this domain is less well defined and is missing from the European directive. It is also susceptible to very different interpretations from one country to another. Academics scored higher for competences related to science and research, *i.e.*, competence 4 (capacity to evaluate scientific data in line with current scientific and technological knowledge) and competence 6 (research).

For cluster 7, community pharmacists posted seven comments. This represented 0.4% of the potential total number of comments (= 258 community pharmacists × 7 propositions in cluster 7). Comments on other propositions were equally low. Comments on cluster 7 concerned the practicality of doing research “*not always practical in a busy community setting*”. Other points raised concerned the access to scientific information from reliable sources “*preselection of the new scientific information by an official institute of continuing education is necessary*” and “*get essential information from reliable sources*”. Academics posted 12 comments (0.7% of potential total) often along the same lines “*impossible for a clinical practitioner to keep up to date with even a small area of therapeutics*” and “*they are able to find synthesised forms of data (meta-analysis, systematic reviews) from trustworthy sources*”. Academics suggested that research concerned advanced studies “*important for more scientifically oriented pharmacists (Ph.D. students)*”.

In cluster 8 (values) academics scored competence 10 (responsibility for patient care) lower than did community pharmacists (88 *versus* 95%). This may be an indication of the lesser weight academics give to patient care. Albeit one academic commented “*I consider a professional approach to patients and their care as the absolute priority*”. Overall there were eight comments from academics and four from community pharmacists. One community pharmacist commented in relation to taking responsibility “*highly depends on how much information we have on patients*”. Other limitations were raised such as that from an academic “*pharmacists can't take responsibility of patient's medication; in Finland this belongs to doctor*s”. Another academic suggested that pharmacists should be able “*whistle-blow and call out poor practice of others*”.

For two competences in cluster 9 (communication and organization) scores for academics and community pharmacists were substantially different. For competence 18 (production of medicines) academics score relatively high (60%), whereas less than half of community pharmacists (43%) considered this important. Scores for competence 23 (entrepreneurship) were the opposite with academics at 46% and community pharmacists at 64%. Comments centered on competence 18 “*no production of medicines, only dispensing*”, and 21 (communication in English) “*knowledge of English not essential, knowledge of local language is essential*”.

Cluster 10 (“competences” 24–37) on the science of medicines was included because the EU Directive [15] lists these 14 subject areas. These are not competences as such but foundations of competences [16]. The inclusion of these subjects provoked substantial misunderstanding with low scores for “competences” concerned with biology (24) and physics (25). Chemistry and analytical chemistry (27 and 28), and pharmaceutical technology (34) were ranked much higher by academics than by community pharmacists. Pharmacology (32) and pharmacotherapy (33) received scores >90% from both academics and community pharmacists.

Cluster 10 received the most comments—11 from academics and eight from community pharmacists. Comments from academics centered on:

- Subjects
  ○ “*the heart of the job is human biology and physiopathology*”
  ○ *Subjects to be added:*
    ▪ pharmaceutical care
    ▪ clinical pharmacy
    ▪ basic clinical knowledge
    ▪ physiopathology
    ▪ social sciences
    ▪ statistics
- Level and job profile
  ○ “*they should also have a background in sciences in general*”
  ○ “*differences may be in the level of knowledge for particular field on the way of professional specialization, not for the pharmacist at the beginning of the career*”
  ○ “*depends if we have to do with a pharmacist in hospital, industry, academy, government or local pharmacies*”

Comments from community pharmacists centered on:

- *Subjects to be added:*
  ▪ Pharmaceutical care
  ▪ business administration
- Level and job profile
  ○ “*being a pharmacist you need the basic knowledge of all the above area. Having a speciality will be important depending the sector you are going to practice*”
  ○ “*all answers refer to daily work in community pharmacy*”

Scores for competences in cluster 11 (industrial pharmacy) were high for academics (< 75%), but lower for community pharmacists (< 65%). There were marked differences e.g., for competence 41 (drug registration): academics 72%, community pharmacists 56%. Comments centered on job profile, with, for example, from academics: “*not really the daily preoccupation of most pharmacists*”, “*some points are essential for pharmacist in pharmaceutical industry, research and development, but it is of little importance for other pharmacists*”, and from community pharmacists “*it's not my job*”. Comments from academics raised the point that all pharmacists should have knowledge of pharmaceutical production as a basis for community practice, e.g., “*must be able to understand the security reasons behind withdrawals to explain them*”. There was some confusion regarding competence 42 (good clinical practice), some applying this to the drug research and development process, and others to community pharmacy practice.

Scores were generally high for clusters 12–19 with only one difference between academics and community pharmacists. This was for competence 53 (familiarity with the bio-pharmaceutical, pharmacodynamic and pharmacokinetic activity of a substance in the body) with academics scoring higher than community pharmacists.

Comments concerned aspects such as diagnosis (competence 44) “one of the challenges that we have come across with extending roles for pharmacists relates to body contact tests and invasive tests. Should these be covered?” Other comments concerned prescription “I would regard it as essential to be able to advise physicians, but currently it is not within the scope of practice for pharmacists to prescribe.”

There were also comments on the level at which specific competence should be taught—“*competences in basic drug contraindications (drug-disease interactions) and knowledge of molecular mechanism of drug-drug interactions must be excellent already when the student finishes the university. Clinical relevance of drug-related problems plus conditions contributing to clinically significant drug-drug interactions, side effects, etc. should be trained on postgraduate level*.”

Scores for competence 57 (manufacture of products not commercially available) were low. Comments centered on the fact that this activity has almost disappeared in most EU countries given the introduction of stringent regulations on GMP: “*manufacturing medicinal production is well under way of near prohibition, with stringent regulations to insure GMP. Both a good point and a bad point, as this means higher quality and controllability, but near industrial manufacturing units in a few selected drugstores, and a specific class of pharmacists*.”

This low score and that for cluster 11 reveals a low awareness amongst community pharmacists that pharmacists are “medicines specialists” involved in the whole drug life cycle from R&D, through production, quality assurance, registration, patient care, pharmaco-economics and post marketing studies. This is not good for the pharmacy profession*.* Taken to extremes it may be concluded—based on the opinion of community pharmacists—that there is no need to employ pharmacists in the medicines industry.

Scores for cluster 16 (patient education) were high. Academics commented that “*pharmacists have a key role in offering public health/healthy living advice*” with the proviso that “*general information on diet or exercise is important but the specific recommendations for the patient should be made by the experts in those areas (f. ex. dietician or physiotherapist)*.”

Scores for cluster 17 (provision of information and service) were high. Comments centered on “*pharmacist should also provide information about medical devices and other items available in the pharmacy*”, and “*need to ensure all information and products are evidence-based and appropriate for that patient*.”

Scores for cluster 18 (monitoring of drug therapy) were high. Academics commented that “*safety and effectiveness are paramount—ensuring we learn from medication errors, post-marketing surveillance and demonstrate clinical effectiveness*.”

Scores for cluster 19 (evaluation of outcomes) were low. Academics commented that these competences were in the domain of clinical or hospital pharmacy.

There were several general comments on methodology, for example, on the use of idiomatic English and phraseology. The construction of proposals with two or more points raised in one question (e.g., for competence 23) also clouded the issue. These and other comments on practicalities have been taken into consideration in the production of the revised version of the questionnaire. Finally, comments throughout pointed to the esoteric nature of certain competences and the need for recognition of specialization.

**Figure 1:**
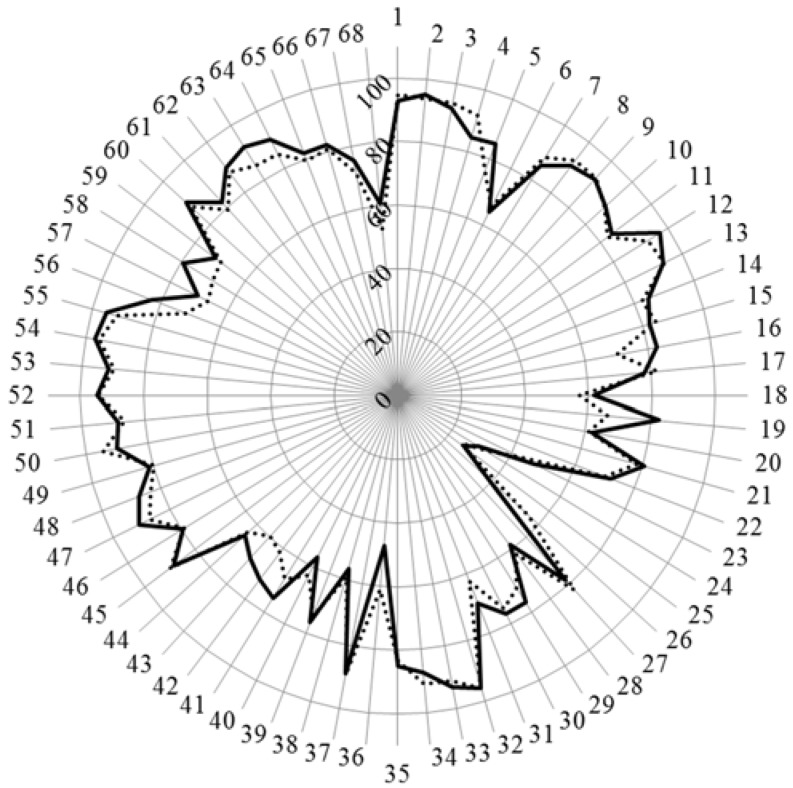
Scores (%) for rankings of competences by academics over 40 (*n* = 144, solid line) and under 40 (*n* = 97, dotted line).

Concerning the age group sub-analysis, the scores for the age subgroups of academics are given in Figure 1.

Chi-square revealed one difference only—that for competence 19 (quality of service) where academics over 40 scored higher than those under 40. For competences 6, 18, 28, 38, and 40 where there were major differences between academics and community pharmacists (see above), there were no significant differences between the two age subgroups of academics. Overall these data suggest that there is no evolution in the opinions, concerning the topics mentioned, of academics with age. Furthermore the fact of having been exposed during their own education to concepts such as “pharmaceutical care” and “competence for practice” (*i.e.*, < 40 years of age) does not appear to influence their rankings for patient care competences. Carrying on from this, it can be suggested that the differences between academics and community pharmacists noted previously is not a question of age.

The above results can be understood very negatively, *i.e.*, what academics learned 25 years ago they consider is still valid. The study is cross-sectional and measures what academics > 40 think today, not what they thought 25 years ago. There is probably no difference with age due to evolution of opinions according to the state of the art in the field: evidence-based pharmacy influences opinions of academics regardless of age.

**Figure 2:**
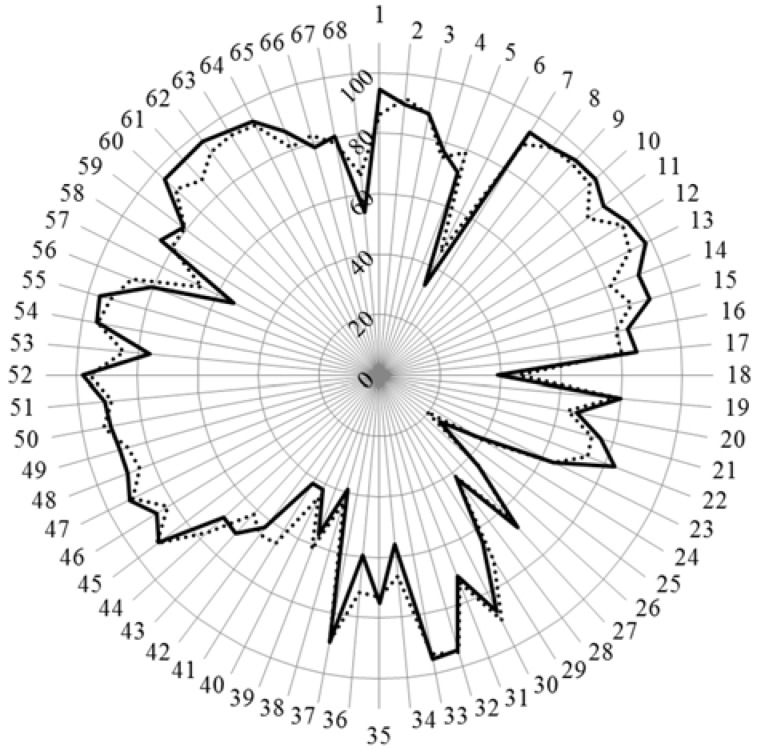
Scores (%) for rankings of competences by community pharmacists over 40 (*n* = 110, solid line) and under 40 (*n* = 148, dotted line).

The scores for the age subgroups of community pharmacists are given in Figure 2.

Chi-square revealed one difference only—that for competence 41 (drug registration) where community pharmacists under 40 scored higher than those over 40; otherwise there were no statistically significant differences. It is to be noted that for the 7–17 competences concerning values and communication, as well as in the group 60–65 concerning again the relation with patients, young pharmacists were somewhat less enthusiastic than their elder colleagues, in spite of the clear movement of community pharmacy practice in this direction. For competences 6, 18, 28, 38, and 40 where there were major differences between community pharmacists and academics (see above), there were no significant differences between the two age subgroups of community pharmacists. Overall these data suggest that there is no evolution in the opinions of community pharmacists with age.

## 4. Conclusions

While surveys of pharmacist perceptions of competence standards have been carried out elsewhere, for example in Thailand [17], to our knowledge, this is the first such survey on a large scale in Europe, although a similar study on competences for medical doctors has been published by MEDINE. Many techniques can be used to identify competencies for practice. Grussing [18] has suggested using multiple methods such as generation by a panel, validation by practitioner surveys or by job analysis. We used the first two of these: generation of a proposal for competences by a panel of experts followed by validation on a large, European-wide scale. We suggest that this double approach strengthens the recommendations of the PHAR-QA consortium.

The general message of this PHAR-QA study is that both academics and community pharmacist recognize the importance of the notion of patient care, as reflected by the high scores for patient care competences. The rankings and the comments underline the nature of the pharmacist as a specialist of medicines, capable making critical evaluations on therapy, and advising the patient as to the best use of medicines.

Community pharmacists gave low scores to drug research, development and production, in spite of the fact that, historically, these domains are the building blocks of pharmacy. As far as research is concerned, this low score is not reflected in the opinions of other healthcare professionals. Thus in the MEDINE2 study on competences for medical practice, medical doctors thought that “*learning outcomes related both to* ‘*using research’ and ‘doing research’ should be core components of medical curricula*”.

In other studies the message is more equivocal. In the Elvey *et al.* [19] study (Who do you Think You Are? Pharmacists’ Perceptions of Their Professional Identity), the authors asked professional pharmacists to give their opinions on nine possible professional identities. They concluded that “While the scientist was the strongest professional identity to emerge it nevertheless seemed to overlap and compete with other professional identities…”

This leads on to the question asked by Waterfield [20]: “*Is pharmacy a knowledge-based profession?*” Waterfield concludes that “*The closer integration of science and practice is another area that needs to be considered by educators as they consider the place of practice-based knowledge*.” They stress the importance of the place of science in pharmacy curricula and practice. The future challenge will be to instill a sense of science-based practice into the mind of the practizing pharmacist, and this via both pre- and postgraduate (continuous professional development) education.

The PHAR-QA study raises the point of specialization in pharmacy education. The nature and depth of the subjects to be dealt with varies as a function of the orientation for a given professional practice. Several comments suggested that competences dealing with research, industrial pharmacy, and pharmaceutical technology belong to specialized, postgraduate education for industrial pharmacy. Competences related to assessment of outcomes and monitoring of patient care; these were considered to be in the domain of a hospital pharmacy specialization.

## 5. Perspectives

In the light of the rankings and comments, a revised version of the survey on the competence framework was produced [21]. This second round will be followed by the publication of a PHAR-QA competence framework for pharmacy practice. Using the PHAR-QA competence framework it will be possible to compare attitudes with the emphases of the same categories in current pharmacy school curricula. Thus suggestions could be made for change in such curricula to address what seems to be a shifting preference of emphasis.

The PHAR-QA competence framework could be incorporated into pharmacy education at various levels of Miller’s triangle [22]. This triangle describes a conceptual, pyramidal model of the various facets of competence with four levels, from level 1 “knows” to level 4 “does”. Given the low scores of most of the subject areas (cluster 10), it would appear that academics and community pharmacists do not grasp the importance of level 1 subjects as building blocks of the level 2 competences. The PHAR-QA framework could be used to develop integrated, coordinated courses that combine several subjects under a broad competence heading. Another use of the PHAR-QA competence framework could be the accreditation at level 2 “knows how”. This would allow a realistic evaluation of a student’s ability to synthesize different subjects into comprehensive competences. The PHAR-QA framework could also be used at the third level in the performance testing of students. Patient substitutes could present students with elements such as symptoms, prescriptions, *etc.* calling upon PHAR-QA competences to solve problems related to drug interaction and other aspects.
